# Fast and Slow Response of the Accommodation System in Young and Incipient-Presbyope Adults During Sustained Reading Task

**DOI:** 10.3390/jcm14041107

**Published:** 2025-02-09

**Authors:** Ebrahim Safarian Baloujeh, António Queirós, Rafael Navarro, José Manuel González-Méijome

**Affiliations:** 1 Institute of Nanoscience and Materials of Aragón (INMA), Spanish National Research Council (CSIC), 50009 Zaragoza, Spain; 2 Department of Applied Physics, University of Zaragoza, 50009 Zaragoza, Spain; 3 Clinical and Experimental Optometry Research Lab (CEORLab), Physics Centre of Minho and Porto Universities (CF-UM-UP), School of Sciences, University of Minho, 4710-057 Braga, Portugal

**Keywords:** autorefractometry, accommodation, presbyopia, sustained reading, peak velocity

## Abstract

**Objectives**: To investigate the dynamics of accommodation during and immediately after a sustained reading task on a digital device across various age groups under monocular and binocular conditions. **Methods**: Seventeen subjects were selected and divided into three age groups: young adults (n = 4, age: 21.3 ± 3.2 years), adults (n = 4, age: 34 ± 3.56 years), and incipient presbyopes (n = 9, age: 45 ± 3.61 years). Dynamic accommodation and disaccommodation were objectively measured using the WAM-5500 open-view autorefractor during 2 min of distance fixation (Maltese cross at 6 m), 5 min of sustained near reading on a teleprompter app at the nearest readable distance, and 2 min of distance vision. Six sequential temporal landmarks were identified. Quantitative metrics for accommodation lag (AL), slope of slow accommodation (SSA), slope of slow disaccommodation (SSD), peak velocity of accommodation (PVA) and peak velocity of disaccommodation (PVD) were obtained as absolute values of spherical equivalent refractive (SER) change. **Results**: SSA, SSD, and AL were significantly and positively correlated with age (ρ = 0.75, 0.73, 0.51, respectively; *p* ≤ 0.038). For subjects under 45 years of age SSA and SSD increased quadratically with age, while for those above 45 years, both SSA and SSD decreased linearly. Linear regression of PVA and PVD with age indicated that the disaccommodation mechanism is faster than accommodation (slope = –0.15 and –0.23, respectively). PVA was significantly faster under monocular than binocular conditions (*p* = 0.124). **Conclusions**: Incipient presbyopes demonstrate a complex response in both accommodation and disaccommodation. The accommodation system responds quickly, but there is also a slower response that can provide up to an additional 1D of accommodative response during sustained near reading tasks. It is hypothesized that the crystalline lens exhibits hysteresis in returning to its unaccommodated state, due to its viscoelastic properties, which means it takes time to relax.

## 1. Introduction

Presbyopia is a universal condition characterized by the progressive decline in the eye’s ability to change its optical power for focusing on near objects. By around 45 years of age, 50% of the population requires an optical prescription for near vision [1]. Besides other anatomical and functional changes, this process involves an increase in the stiffness of the crystalline lens due to biochemical alterations [2].

Presbyopia is regarded as one of the most common refractive errors globally. With the world population aging, the prevalence of presbyopia is anticipated to increase markedly. A study conducted in 2018 revealed that around 1.8 billion individuals were presbyopic [3], while the World Health Organization advises this figure will to rise to 2.1 billion by 2030. The effect of presbyopia on an individual’s standard of living is significant. It may make routine near-vision tasks difficult. Additionally, driving and other activities requiring a quick shift between near and far vision are generally more difficult for presbyopes as well. This may lead to minished productivity, especially in low-income nations where up to 94% of cases remain untreated [4].

Current visual habits include continuous exposure to near and intermediate visual demands, followed by periods of distance vision. In a young, fully functional accommodating system, it is reported that the crystalline lens can change its shape in a fraction of a second to a few seconds (depending on differences in the methodology and analysis used to measure response time) to increase or decrease its optical power [5,6]. This allows the human visual system to shift focus from far to near and from near to far without noticeable changes. Instead, young subjects with accommodative dysfunctions, such as accommodative spasm or inflexibility [7], as well as incipient-presbyopic subjects experience transient blurring of vision when looking at distance after prolonged near visual effort. In young eyes, this symptomatology is the manifestation of the ciliary muscle’s difficulty relaxing. This creates tension in the zonular fibers, making the crystalline lens reshape back to its distance power [8]. However, in older eyes, this is likely to be related to increased stiffness of the crystalline lens structure [9,10]. This phenomenon presents an opportunity to investigate the biomechanical properties of the eye through continuous measurement of the optical power changes during and after prolonged exposure to a demanding near vision task.

Previous studies have demonstrated the different responses of younger and older eyes for accommodating stimuli. However, most of these studies have focused only on short-term responses (few seconds), revealing changes in the rapid accommodation and disaccommodation responses at various ages [11,12,13]. By comparing the aging dynamics of far-to-near and near-to-far accommodation in 20 subjects aged 15 to 55 years, Beers and Van der Heijde proposed a mechanical model of the accommodation mechanism, simply using springs and a dashpots [11]. Kasthurirangan et al. investigated how the dynamics of accommodation and disaccommodation depend on response amplitude [14]. Lockhart et al. employed a customized autorefractor to examine changes in accommodative dynamics as a function of age [15]. Kasthurirangan and Glasser also showed that the eye’s ability to accommodate and disaccommodate changes with age [16].

Despite intensive research in the field of dynamic accommodation, to the best of the authors’ knowledge, no previous study has systematically investigated the response during a period of several minutes of near visual effort and the subsequent recovery.

This work hypothesizes that it is possible to demonstrate the existence of a slow response that follows the fast response typically measured in accommodating tests, whether in clinical setting (amplitude of accommodation, accommodation flexibility) or in research context (short-term dynamic accommodation tests). This slow response in accommodation and relaxation/disaccommodation would be able to indirectly demonstrate the progressive changes in the accommodation system, highlighting the difficulties the crystalline lens faces in changing its shape.

Therefore, the primary goal of this study was to correlate the parameters extracted from the refractive power changes of the eye during and after a period of several minutes of near vision effort in healthy subjects of varying ages, from young adults to pre-presbyopes. The methodology presented here is expected to be used to indirectly characterize the optomechanical properties of the human crystalline lens and enhance our understanding of its aging process.

## 2. Methods

### 2.1. Materials

The Grand Seiko WAM-5500 open-view autorefractor (Grand Seiko Co., Ltd., Hiroshima, Japan) was used in this study in high-speed mode to dynamically measure the spherical equivalent refractive responses of the eyes, which can be interpreted as accommodative and disaccommodative responses. The autorefractor was connected to a computer via an RS232 port, and a custom software (DRER, CEORLab, Braga, Portugal) was employed to continuously record the SER value of the eye in a CSV file. The measurement principle involves projecting an infrared ring of light onto the retina. Then, the ring’s image, which is reflected off the retina, is sharpened by rapidly moving a lens along a motorized track. The size and shape of the reflected ring determine the sphere, cylinder, and axis [17]. According to the manufacturer, this device can measure refraction within ±22 D of sphere and ±10 D of cylinder (in steps of 0.01, 0.12, or 0.25 D), and angles from 0 to 180° (1° steps). Nevertheless, only the SER value is recorded by the device while operating in dynamic mode. Blinks, movements, or measurement errors can result in missing SER values in the output file; these were replaced by adjacent values later. To compensate for the small eye movements of the subject during sustained measurements, the operator actively used a joystick to adjust the autorefractor’s head horizontally and vertically to keep the circle as centred and as focused as possible.

A Galaxy Tab S9 Ultra tablet (Samsung Electronics Co., Ltd., Suwon, Republic of Korea) was used as the near target in this experiment, but only an area limited to 2.5 × 3 cm^2^ of its screen was exposed to the eye. An online teleprompter was loaded on the tablet to display a text (white characters on a black background, font Arial 12) in the region of interest, while automatically scrolling at a predefined speed (1.6 lines/s) to minimize eye movements, so the autorefractor could continuously record the SER of the eye. A table was mounted over the autorefractor without restricting its movement, allowing the tablet to be positioned in front of its hot mirror for the sustained near vision task. The nearest readable point was marked in advance for each subject on the table, and the tablet was placed or removed as soon as the far and near tasks were completed.

### 2.2. Subjects and Experimental Conditions

Twenty-four healthy volunteers were recruited for this study, using their current prescription if they were not emmetropic. The following exclusion criteria were established: presence of ocular disease, being fully presbyopic (showing no accommodative response at near), or inability to fixate or to maintain attention throughout the experiment. The remaining seventeen subjects were divided into three age groups: under 30 years (young adults, 4 subjects, mean age 21.3 ± 3.2), 30–39 years (adults, 4 subjects, mean age 34 ± 3.56), and 40–50 years (incipient presbyopes, 9 subjects, mean age 45 ± 3.61). If a subject was unable to maintain attention or keep their head and eye steady during one of the measurements and their SER could not be continually recorded, we still considered the correctly measured eye in the analysis, which accounts for the difference in sample sizes.

Besides, some were removed due to difficulties in maintaining attention or steady head and eye positions throughout the experiment, preventing continuous recording of their SER.

**Figure 1:**
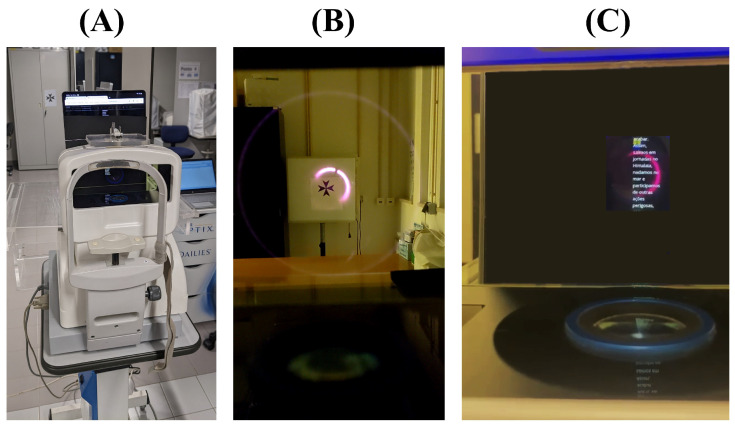
Experiment in action (**A**). Far (**B**) and near (**C**) targets are aligned inside the measuring infrared ring of the autorefractor to reduce measurement error.

To assess accommodative and disaccommodative responses, participants were instructed to rest their chins in the chinrest of the open view autorefractor and follow these steps, once in one eye, then in the other, and then binocularly (but as will explain later, a random eye from each subject was selected for subsequent analyses):

- They looked through the autorefractor hot mirror at a far target (Maltese cross measuring 10 × 10 cm^2^, placed 6 m away, 25.37 cd/m^2^, 29.7 lux) for 2 min; ensuring that the far target was visible within the measuring infrared circle (Figure 1A).
- Immediately afterward, the region of interest on the tablet was positioned aligned with the eye at the nearest readable distance to the subject, ranging from 17 cm (31.35 cd/m^2^, 2.82 lux) to 41 cm (31.35 cd/m^2^, 9.38 lux). This distance was carefully selected to induce maximum accommodative demand, with a maximum upper limit of 5.88 D. Subjects read an autoscrolling text within this region for 5 min, ensuring that the text remained aligned with the measuring infrared circle (Figure 1B).
- Subsequently, the tablet was removed, and subjects shifted their focus back to the far target for another 2 min (Figure 1A). Maintaining alignment of the targets within the measuring infrared circle was emphasized, as it is crucial for the accuracy of optical measurements [18,19].

### 2.3. Data Processing

The exported CSV files were analyzed using MATLAB R2023a software (MathWorks, Inc., Natick, MA, USA) in a semi-automatic manner. To the best of the authors’ knowledge, the dynamics of accommodation have not been studied under sustained demands for accommodation and disaccommodation, with recorded responses typically limited to a few seconds. However, identifying critical points, such as the onset of accommodation or disaccommodation on the response graph and the analyzing strategy have always been challenging, especially since they affect the results, and this study allows for comparison with existing literature. Exponential and sigmoid functions were employed to fit the accommodative and disaccommodative responses. Smoothing the data using the Savitzky–Golay method is another method used by researchers to study the dynamics of accommodation [20]. Finding parameters such as reaction time by defining the time limited to 80% of the accommodation response is another example of user-defined criteria [21]. Nevertheless, manual selection of points on the response graphs remained the common approach [15], as Lockhart and Shi stated by reviewing research studies [22,23]. Since we did not intend to study time-related parameters, such as time constants, determining the points which enabled us to estimate the fast accommodation/disaccommodation responses, and subsequently the slopes of slow accommodation/disaccommodation, were the most important points. We calculated the slopes initially using these points, followed by an automatic linear fitting method, and compared the results to identify any differences.

**Figure 2:**
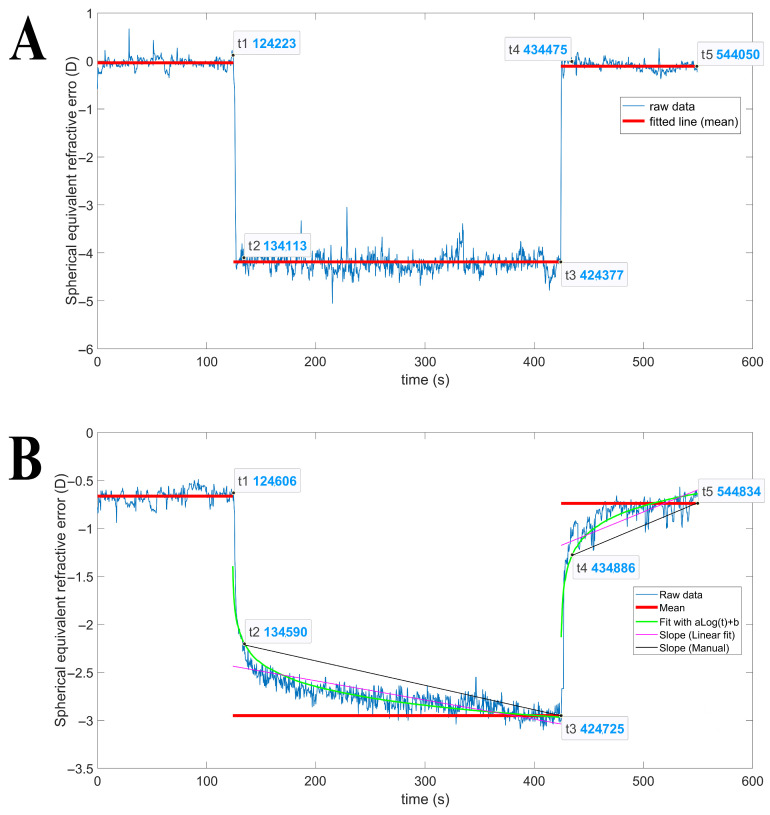
Far-to-near and near-to-far sustained accommodation/disaccommodation responses in a young-adult (**A**) and in an incipient presbyope (**B**).

Looking at Figure 2, the time points of t_1_-t_5_ were manually selected, and the software calculated various parameters based on these selections. The t_1_ point represents the onset of accommodation, marking the moment when subjects shifted their focus from the far target to the near target (120 s from t_0_ to t_1_). The mean SER value in this t_0_-t_1_ period was considered the magnitude of relaxed accommodation. The t_2_ point was designated as (t_1_ + 10 s) and named fast accommodation response time, because after 10 s from the onset of accommodation, subjects were able to adapt to autoscrolling speed and smoothly follow the text on the tablet’s region of interest. By this time, accommodation overshootings had subsided, allowing us to consider the average time taken to place the near stimulus in front of the eyes. Of note, peak velocities occur within this 10-s window.

Based on these graphical results, choosing the SER value at t_2_ posed a challenge since the young crystalline lens could accommodate quickly in a step function, while the old lens followed a logarithmic adjustment for accommodation (green trend). As a result, when the accommodation response deviated from the logarithmic function, the SER value from the smoothed raw data was used for parameter calculations. Similar to t_1_, t_3_ point was also considered as the onset of disaccommodation, when subjects shifted their focus from the near target to the far target (300 s from t_1_ to t_3_). The maximum accommodation magnitude is calculated as the average within the last minute prior to t_3_ since the accommodation curve usually reaches its maximum around the last minute of near work, so accommodation response (AR) was calculated by subtracting the magnitude of relaxed accommodation from this value. Accommodation lag (AL) was also estimated as the difference between the accommodation demand of the near stimulus and the AR. By drawing a line between t_2_ and t_3_, the slope of slow accommodation (SSA) could be estimated (black line). SSA can also be calculated through linear fitting of the accommodation response within the t_1_–t_3_ window (purple line). Following a similar argument and way to t_2_, t_4_ was selected as (t_3_ + 10 s) and designated as the fast disaccommodation response. By selecting t_5_ as (t_3_ + 120 s), the slope of a line crossing t_4_ and t_5_ (black line) can be estimated and called the slope of slow disaccommodation (SSD). SSD can similarly be calculated by linear fitting of the disaccommodation response in the t_3_–t_5_ window (purple line).

**Figure 3:**
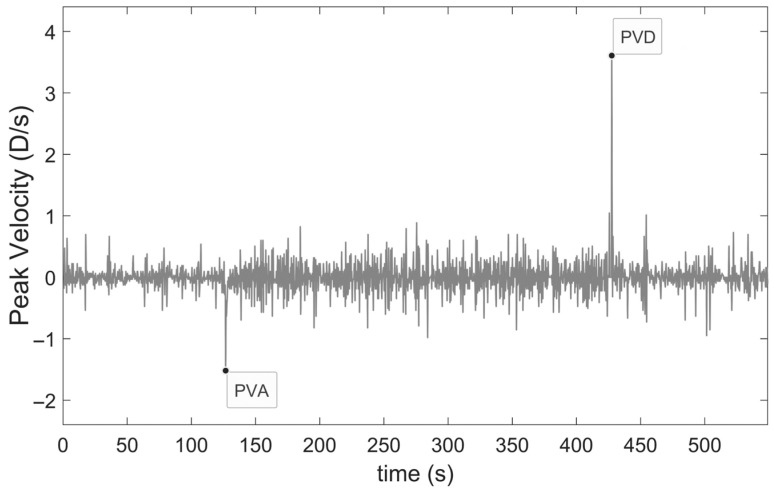
Peak velocity of accommodation (PVA) and peak velocity of disaccommodation (PVD) during measurement for an incipient presbyope.

Taking the derivative of the SER raw data (Figure 2) produces the velocity dynamics graph (Figure 3), and by seeking the minimum value between t_1_ and t_2_ and finding the maximum value among t_3_ to t_4_ yields peak velocity of accommodation (PVA) and peak velocity of disaccommodation (PVD), respectively.

Quantitative metrics for accommodation lag (AL), slope of slow accommodation (SSA), slope of slow disaccommodation (SSD), peak velocity of accommodation (PVA), and peak velocity of disaccommodation (PVD) were obtained as absolute values of spherical equivalent refractive (SER) change.

### 2.4. Data Analysis

Statistical analysis was conducted using SPSS 25 (SPSS Inc., IBM Corporation, Somers, NY, USA) and Excel 2019 (Microsoft Corporation, Redmond, WA, USA). Since the data were not normally distributed, the following nonparametric methods were employed: Spearman’s correlation test to assess the correlation between parameters such as SSA, SSD, PVA, PVD, AL, and AR with age. Spearman’s rho parameter (ρ) is reported. Kruskal-Wallis test, together with a post-hoc pairwise comparison test, was applied to compare the above parameters in our age groups. Two-sample Wilcoxon test was used to compare the above-mentioned parameters in binocular and monocular measurements.

## 3. Results

A Spearman’s correlation was conducted to evaluate if there is a correlation between accommodation and disaccommodation parameters of the subject’s right and left eyes (Table 1). Out of seventeen subjects, three had extensively moved their eyes or heads during measurement of one of their eyes, resulting in data from only fourteen subjects being suitable for comparison. Significant correlations were found between the right and left eyes for all measured parameters. Correlations ranged from 0.591 (PVD, *p* = 0.026) to 0.991 (AR, *p* < 0.001). Therefore, a random eye from each subject was selected for subsequent analyses.

Once again, Spearman’s correlation analysis was conducted to evaluate the relationship between accommodation and disaccommodation parameters and age across all eyes (Table 2). Parameters of SSA, SSD, and AL showed significantly positive correlations with age (ρ = 0.75, 0.73, 0.51, respectively; *p* ≤ 0.038 for all). There were also significantly negative relationships between the PVA, PVD, and AR with age (ρ = −0.91, −0.74, −0.93, respectively; *p* < 0.001 for all).

**Figure 4:**
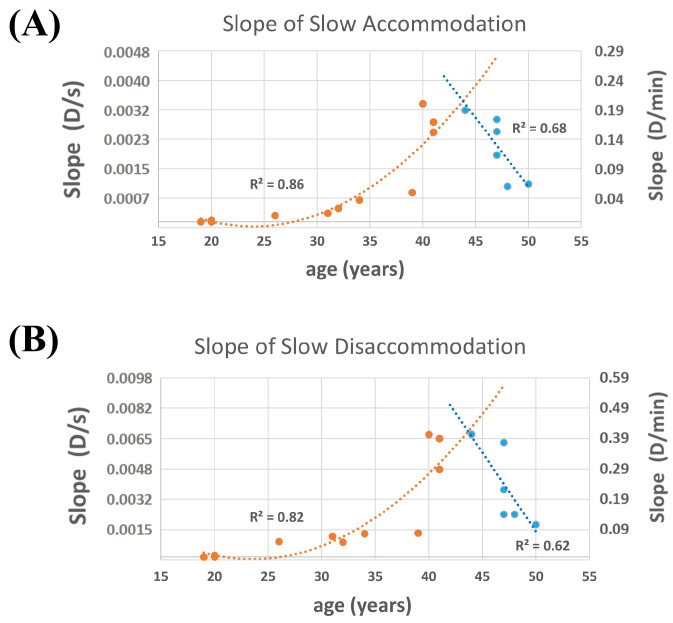
Quadratic and linear regression of slope of slow accommodation (**A**) and slope of slow disaccommodation (**B**), before (orange dots) and after age of 45 (blue dots).

When considering SSA and SSD separately for subjects under and above 45 years of age, we could still employ regression methods to illustrate the relationship between these parameters and age (Figure 4). The analysis revealed that both SSA and SSD changed quadratically up to age 45 (R^2^ = 0.86, *p* < 0.001; and R^2^ = 0.82, *p* < 0.001, respectively) and decrease linearly afterward (R^2^ = 0.68, *p* = 0.045; and R^2^ = 0.62, *p* = 0.064, respectively). We calculated SSA and SSD once using the visual selection of critical points and then in an automatic linear fitting method. Notably, while the visual method showed significant correlations with age, the automatic method did not reveal any correlations (*p* > 0.05), confirming the superiority of our proposed visual method.

**Figure 5:**
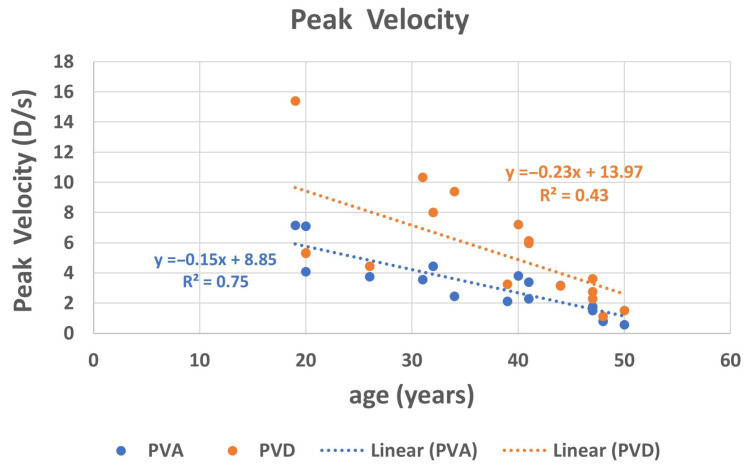
Change in peak velocity of accommodation (PVA) and peak velocity of disaccommodation (PVD) with age.

Analyzing PVA and PVD in relation to age using linear regression (Figure 5) indicates that the disaccommodation mechanism is faster than accommodation (slope = −0.15 and −0.23, respectively; R^2^ = 0.75 and 0.43, respectively; *p* ≤ 0.004 for both).

**Figure 6:**
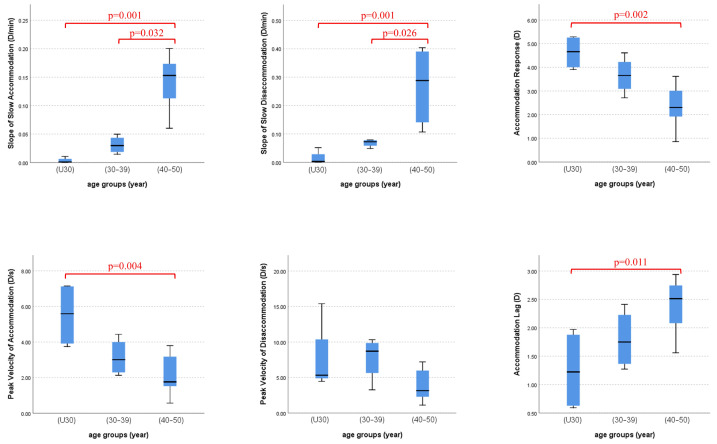
Independent samples Kruskal–Wallis test results for finding differences in accommodation/disaccommodation parameters among our age groups. U30 denotes eyes belonging to subjects under 30 years of age. The group (30–39) represents adults, and the group (40–50) denotes incipient presbyopes. *p*-values for statistically significant differences among group pairs are reported on graphs in red.

A Kruskal-Wallis test with post-hoc pairwise comparison was conducted to identify differences in accommodation and disaccommodation parameters among our age groups. Statistically significant differences were observed between age and: SSA (H(2) = 13.27, *p* = 0.001), SSD (H(2) = 12.98, *p* = 0.002), PVA (H(2) = 8.52, *p* = 0.014), PVD (H(2) = 5.14, *p* = 0.077), AR (H(2) = 10.32, *p* = 0.006), and AL (H(2) = 7.34, *p* = 0.025). Pairwise comparisons (Figure 6) revealed which pair of groups have statistically significant differences among themselves, indicated with *p*-values on the graphs.

Accommodation and disaccommodation parameters were also measured binocularly, and a Wilcoxon signed-rank test revealed that performing the designed experiment binocularly did not elicit a statistically significant difference among the parameters, compared to monocular measurements, except for the PVA parameter (z = −1.54, *p* = 0.12) (Table 3).

In other words, performing the task binocularly resulted in a higher peak velocity of accommodation than monocularly. Table 4 presents the descriptive statistics of the performed measurements.

## 4. Discussion

Despite centuries of investigation into the phenomenon of accommodation, its underlying mechanisms are still not fully understood. To the best of the authors’ knowledge, this study is the first to use an autorefractor for sustained near-to-far and far-to-near tasks to investigate changes in crystalline lens power during measurements. Previous studies have considered short-term accommodation responses with task durations of a few seconds in order to understand the fast response of the accommodation system during changes to near and distance targets [14,20,21,24]. This paradigm of research has proven effective for evaluating the accommodative response to fast changes in distance viewing. Such a fast response is present in young eyes where the accommodation system can be characterized by an elastic behaviour with fast accommodation and fast recovery of the optical power of the crystalline lens. However, it does not necessarily reflect the actual capabilities of the accommodation system in older eyes, where biological changes in the crystalline lens can increase its viscosity, resulting in slower accommodation and disaccommodation responses. These changes have been highlighted in the findings of Kasthurirangan and Glasser, who reported a different path of optical power changes during 6 s of accommodation between young and adult eyes [16]. Several clinical observations suggest that as the eye ages, the ciliary muscle encounters increasing difficulty in reshaping the crystalline lens. For instance, some presbyopic patients report deterioration in image quality immediately following a sustained near vision task, with subsequent improvement in image quality noted 5 to 10 min later [25]. Rather than an actual change in the optical power of the eye, this has been explained as an adjustment of the viewing distance, and/or blur adaptation to defocus. On the other side, some incipient presbyopes commonly experience transient blurred vision after a sustained near vision task. Besides the explanations, this may be attributed to the lens’s slower ability to reshape back after being subjected to a power change for near focus. Recent studies conducted by the authors of the present work revealed that higher-order aberrations increased following a 5-min near vision reading task [26]. A subsequent study [25] demonstrated that the eye’s image quality undergoes changes 10 min after a sustained reading task. In contrast to previous studies, this work evaluated the slow response of the accommodation system in real time, continuously collecting refractive data over a total duration of 9 min, which included 5 min of sustained near reading activity. Therefore, we present a substantially different research paradigm from those previously described in the literature on accommodation and disaccommodation.

In the present study, young subjects quickly reach their maximum level of accommodation and maintain that level while reading. In contrast, incipient presbyopes can gradually increase their accommodation magnitude over time, achieving a higher value during a 5-min effort, as noted in our pilot study. The same applies to the process of accommodation relaxation, where the crystalline lens exhibits hysteresis in reshaping back to its unaccommodated state due to its viscoelastic properties, requiring time to relax. Mordi and Ciuffreda demonstrated that the amplitude of accommodation decreases with age [27], which aligns with our findings of decreased accommodation response (AR) and increased accommodation lag (AL) with age. Lockhart and Shi also reported that PVA and magnitude of accommodation tend to decline with age [15]. Schaeffel et al. found that the disaccommodation process was quicker than accommodation [28]. We confirmed both by analyzing PVA and PVD. Although Kasthurirangan and Glasser reported that a decreased in peak velocity with age, their assertion that the peak velocity of disaccommodation does not decrease with age contrasts with our results [16]. This discrepancy might be related to the methodology they used to estimate velocity. They concluded that the ciliary muscle and body play a smaller role in presbyopia development compared to the crystalline lens and capsule. Based on their mechanical model, Beers and Van der Heijde stated that the viscoelastic aspect of the lens mostly determines the far-to-near time constant, whereas the elastic properties of the zonules and choroid, together with the viscoelastic properties of the lens, play a role in the near-to-far time constant [11].

Comparing binocular and monocular measurements, we found no significant differences in accommodation and disaccommodation parameters, except for PVA. This aligns with the findings of Chirre et al., who reported a faster accommodation response under binocular viewing conditions [24]. With the introduction of the metrics for SSA and SSD, we were able to quantitatively compare the slow accommodation and disaccommodation responses. Two methods were proposed for estimating them; the visual method yielded better results than automatic linear fitting for analysing sustained accommodative dynamics. Analysis of SSA and SSD with age showed consistency with the findings of Pellegrino et al., who noted the delay in reshaping the lens to its initial state in partially presbyopic lenses [29]. We observed a quadratically increase in SSA and SSD up to age 45, followed by a linear decrease. This may be attributed to the reduced amplitude of accommodation in incipient presbyopes, which lowers the slope of these responses.

By recalculating and reinterpreting the data from Heys et al. [30], Augusteyn suggested that the stiffness of the nucleus and cortex of lens change with age in a sigmoidal manner, with most changes occurring between ages 30 and 50 [31]. Although stiffness of the lens was not studied, our results suggest a potential relationship with stiffness changes (Figure 4), as both SSA and SSD quadratically increase with age until 45, followed by a linear decrease, since accommodation/disaccommodation response tends to be zero in fully presbyopic eyes. When the slope of slow accommodation is decreasing (after age of 45), it means that the accommodation response is lower than it was before this age, likely due to increased stiffness. In other words, we indirectly demonstrated the biomechanical behavior of the lens using SER change in our experiment. Despite this, we cannot assume a cause-effect relationship.

Moreover, by studying the electrical impedance of the ciliary muscle during accommodation, Swegmark demonstrated that the ciliary muscle continues to function at least until the age of 60 [32]. Fisher later reported that the maximum force of contraction exerted by the entire ciliary muscle increases from ages 30 to 45, then decreases by age 60 [33]. Burd et al. constructed a finite element model to estimate the force acting on the lens and its changes with age during accommodation [34]. They reported a pattern similar to Fisher’s results [33], though with greater significance. Although we did not study the ciliary muscle changes, our results may suggest a relationship with changes in the applied force of ciliary muscle (Figure 4), as with change of maximum contractile force of ciliary muscle by age, accommodation/disaccommodation response and, subsequently, SSA and SSD change with age.

From a biochemical standpoint, lens proteins undergo chemical alterations that may increase lens stiffness and lead to presbyopia. Lens disulfides demonstrate a significant increase with age, while glutathione levels negatively correlate with age. Garner and Garner treated mouse lenses with R-lipoic acid, reporting a reduction in protein disulfides and a subsequent decrease in stiffness [35]. Meanwhile, Nandi et al. believed in other factors, such as the role of dicarbonyl-mediated advanced glycation end products (AGEs) formation in lens stiffening, and developed the carboxitin molecule to treat bovine and mouse lenses with it [36]. These age-related biochemical changes in the lens correlate with a reduction in the accommodation response with age, as reported in this study.

The present study had some limitations. The reduced sample size in ophthalmic studies presents a significant challenge to the generalizability of the findings. A reduced sample may not accurately represent the diversity of the full spectrum of the population, which can be critical when dealing with a range of age groups. Additionally, a small sample size increases the margin of error, making the results more prone to variability and reducing the precision of conclusions regarding parameters. To improve the precision of future research, it is important to aim for larger and more representative sample sizes.

Another limitation of the study was related to the measurement method used. Several individuals were excluded from the analyses because they were unable to adhere to the specific measurement protocol, making the sample size smaller. To address this issue in future studies, improving the clarity and comprehensiveness of the instructions could be crucial. By providing more detailed guidance and potentially offering additional support to participants, it would increase the likelihood of adherence to the protocol, ensuring a more representative sample and more robust results. Furthermore, incorporating a feedback mechanism or reminders throughout the measurement process could help mitigate errors related to protocol non-compliance.

Future studies should systematically investigate the slow changes in the power of the crystalline lens to establish normative parameters for this process in a representative population sample. Repeating this research using an open-view OCT or Double-pass system [37] could provide supplementary insight with dynamic changes in parameters such as axial length, anterior chamber distance, and lens thickness, during accommodative/disaccommodative tasks. Investigating the possible differences of accommodation/disaccommodation parameters between dominated and non-dominated eye can be considered in future works as well. This way, future studies could provide a more comprehensive understanding of binocular vision dynamics.

## 5. Conclusions

In conclusion, the present work demonstrates that, besides the fast response of the accommodation system, there is a slow response. This suggests that accommodation dynamics are more complex than previously assumed, especially for incipient presbyopes. Evaluating this complex response, which may involve hysteresis, is crucial for understanding the changes in the accommodation system as the eye ages. While this slow change in the optical power of the eye is not useful for the physiological need during the change of eye focus which takes a fraction of a second, it helps explaining clinically common complaints experienced by presbyopes during or after a sustained near vision task.

## Figures and Tables

**Table 1:** Spearman’s correlation coefficients for accommodation/disaccommodation parameters of the right and left eyes of subjects (sample size: 14).

Parameter 1	Parameter 2	Correlation Coefficient	*p*-Value
SSA_right_	SSA_left_	0.952	<0.001
SSD_right_	SSD_left_	0.934	<0.001
PVA_right_	PVA_left_	0.906	<0.001
PVD_right_	PVD_left_	0.591	0.026
AR_right_	AR_left_	0.991	<0.001

SSA, slope of slow accommodation; SSD, slope of slow disaccommodation; PVA, peak velocity of accommodation; PVD, peak velocity of disaccommodation; AR, accommodation response.

**Table 2:** Spearman’s correlation coefficients for accommodation/disaccommodation parameters of monocular measurements and age (sample size: 17).

	SSA	SSD	PVA	PVD	AR	AL
Correlation Coefficient	0.748	0.728	−0.904	−0.736	−0.925	0.506
*p*-value	0.001	0.001	<0.001	0.001	<0.001	0.038

SSA, slope of slow accommodation; SSD, slope of slow disaccommodation; PVA, peak velocity of accommodation; PVD, peak velocity of disaccommodation; AR, accommodation response; AL, accommodation lag.

**Table 3:** Wilcoxon signed-rank test results for finding differences in accommodation/disaccommodation parameters while performing the experiment monocularly and binocularly (sample size: 17).

	SSA_b_–SSA_m_	SSD_b_–SSD_m_	PVA_b_–PVA_m_	PVD_b_–PVD_m_	AR_b_–AR_m_	AL_b_–AL_m_
Z	−0.170 ^p^	−0.227 ^n^	−1.539 ^n^	−0.213 ^p^	−0.828 ^n^	−0.828 ^p^
Asymp. Sig. (2-tailed)	0.865	0.820	0.124	0.831	0.408	0.408

_b_ Binocularly. _m_ Monocularly. ^p^ Based on positive ranks. ^n^ Based on negative ranks. SSA, slope of slow accommodation; SSD, slope of slow disaccommodation; PVA, peak velocity of accommodation; PVD, peak velocity of disaccommodation; AR, accommodation response; AL, accommodation lag.

**Table 4:** Descriptive statistics of the performed measurements.

	Young Adults	Adults	Incipient Presbyopes	All Participants
Sample size	N = 4	N = 4	N = 9	N = 17
Age (years) Mean ± std	(19–29) 21.3 ± 3.2	(30–39) 34 ± 3.56	(40–50) 45 ± 3.61	(19–50) 36.8 ± 10.53
# Female	3	3	3	9
Measures of accommodation: (Mean ± std)	monocular binocular			
Accommodative response—AR (D)	4.63 ± 0.73 4.58 ± 0.68	3.66 ± 0.79 3.85 ± 0.67	2.33 ± 0.99 2.37 ± 0.91	3.18 ± 1.3 3.24 ± 1.25
Accommodative lag—AL (D)	1.25 ± 0.73 1.31 ± 0.68	1.78 ± 0.53 1.6 ± 0.55	2.4 ± 0.47 2.36 ± 0.55	1.99 ± 0.71 1.93 ± 0.72
Slope of slow accommodation—SSA (D/min)	0 ± 0.01 0.01 ± 0.02	0.03 ± 0.02 0.04 ± 0.02	0.14 ± 0.05 0.12 ± 0.04	0.08 ± 0.07 0.08 ± 0.06
Slope of slow disaccommodation—SSD (D/min)	0.01 ± 0.02 0.02 ± 0.02	0.07 ± 0.01 0.08 ± 0.04	0.27 ± 0.12 0.27 ± 0.12	0.16 ± 0.15 0.16 ± 0.14
Peak velocity of accommodation—PVA (D/s)	5.52 ± 1.86 6.17 ± 0.50	3.15 ± 1.05 3.47 ± 0.60	2.12 ± 1.14 2.59 ± 1.8	3.16 ± 1.87 3.61 ± 1.96
Peak velocity of disaccommodation—PVD (D/s)	7.62 ± 5.20 8.67 ± 2.22	7.75 ± 3.14 6.33 ± 1.06	3.74 ± 2.18 3.51 ± 2.65	5.6 ± 3.66 5.39 ± 3.09

## Data Availability

The data presented in this study are available upon request from the corresponding author.
